# Coding of procedures documented by general practitioners in Swedish primary care-an explorative study using two procedure coding systems

**DOI:** 10.1186/1471-2296-13-2

**Published:** 2012-01-09

**Authors:** Anna Vikström, Maria Hägglund, Mikael Nyström, Lars-Erik Strender, Sabine Koch, Per Hjerpe, Ulf Lindblad, Gunnar H Nilsson

**Affiliations:** 1 Department of Neurobiology, Care Sciences and Society, Center for Family and Community Medicine, Karolinska Institutet, SE-141 83 Huddinge, Sweden; 2 Department of Learning, Informatics, Management and Ethics, Health Informatics Centre-(HIC), Karolinska Institutet SE-171 77 Stockholm, Sweden; 3 Department of Biomedical Engineering, Linköpings University, SE-581 85 Linköping, Sweden; 4 R&D Centre, Skaraborg PC, Skövde, Sweden 5 Social Epidemiology, Department of Clinical Sciences, Faculty of Medicine, Lund University, Sweden; 5 Department of Public Health and Community Medicine/Primary Health Care, the Sahlgrenska Academy at the University of Gothenburg, Sweden

## Abstract

**Background:**

Procedures documented by general practitioners in primary care have not been studied in relation to procedure coding systems. We aimed to describe procedures documented by Swedish general practitioners in electronic patient records and to compare them to the Swedish Classification of Health Interventions (KVÅ) and SNOMED CT.

**Methods:**

Procedures in 200 record entries were identified, coded, assessed in relation to two procedure coding systems and analysed.

**Results:**

417 procedures found in the 200 electronic patient record entries were coded with 36 different Classification of Health Interventions categories and 148 different SNOMED CT concepts. 22.8% of the procedures could not be coded with any Classification of Health Interventions category and 4.3% could not be coded with any SNOMED CT concept. 206 procedure-concept/category pairs were assessed as a complete match in SNOMED CT compared to 10 in the Classification of Health Interventions.

**Conclusions:**

Procedures documented by general practitioners were present in nearly all electronic patient record entries. Almost all procedures could be coded using SNOMED CT.

Classification of Health Interventions covered the procedures to a lesser extent and with a much lower degree of concordance. SNOMED CT is a more flexible terminology system that can be used for different purposes for procedure coding in primary care.

## Background

Primary care in Sweden, with general practice as the core medical specialty, provides ambulatory and home health care outside hospitals. It is regarded as a fundamental constituent of the health care system in Sweden [1] and accounts for 17% of the net costs of health care in the country [2].

Different professionals such as general practitioners (GPs), district nurses, physiotherapists and occupational therapists work in primary care and are required by Swedish law to carry out health care documentation, which is normally done in electronic patient records (EPRs). Health care documentation in Swedish primary care is often considered poorly organized and difficult to use for secondary purposes such as research and follow-up, or for sharing between different EPRs [3]. Narrative, free-text documentation in EPRs is still common.

Structuring and coding of information in EPRs in primary care has been suggested to hold great potential. It may provide a source for research and statistical analysis [4], make an important contribution towards interoperability of health care information systems, and potentially provide a basis for reimbursement models [5]. Extensive work has been performed regarding coding of health care problems and diagnoses in primary care [6-8]. Coding of treatments, prescriptions and referrals by GPs in primary care has also received some attention. Despite this, there is a lack of research describing which procedures are actually performed and how they are documented in primary care EPRs by different groups of health care professionals.

### Procedure coding systems

Numerous national procedure coding systems (PCS) are in use but international standards are generally lacking in the field of medical procedure coding [9]. PCS are often used in connection with reimbursement systems but can also be used for clinical, research and follow-up purposes [9-11]. In this article we refer to PCS as a type of terminology system. Different generations of terminology systems have been described [12]. The first generation is non-hierarchical with a list of phrases, a list of codes and a coding scheme, and often has limitations concerning reuse of coded data. Second generation terminology systems are compositional systems [12] with a knowledge base to define and extend the concepts. The third generation comprises formal terminology systems with a set of symbols and formal rules [12]. In second and third generations of terminology systems, reorganisation of concepts is supported by the knowledge base that defines and extends the concepts and/or formal rules, whereas in first generation systems reorganisation has to be done manually [12]. This is important regarding the ability to aggregate data for different purposes. The content of a terminology system is also of great importance [13] in a multi-professional perspective. Different health care professionals performing procedures in a specific domain, such as primary care, must be able to code the procedures on a sufficient level of detail.

By tradition, procedure coding has been performed to some extent with the International Statistical Classification of Diseases (ICD-10), as ICD-10 primarily includes procedures in Chapter 21 [8]. International Codes in Primary Care (ICPC) is also used for procedure coding in primary care [14].

### Use of Procedure Coding Systems in Swedish Primary Care

In 1998 a PCS for primary care was developed in Sweden based on ICPC [15], but implementation was very limited. Since 2007 it has been mandatory in almost all of health care to report coded procedures to a National Patient Register. However, it is not mandatory to report procedures in primary care, and GPs in Sweden do not normally code procedures [5]. PCS are not systematically used for reimbursement reasons in Swedish primary care.

Currently, two terminology systems are available for procedure coding in Sweden, the national Classification of Health Interventions or KVÅ (Swedish acronym for Klassifikation av vårdåtgärder), which is in present use, and the international SNOMED CT, which has recently been translated to Swedish but is not in common use. SNOMED CT has a broad coverage of topics including procedures/interventions. KVÅ is a relatively new classification, nationally developed and maintained by the National Board of Health and Welfare in Sweden for use by different health care professionals in all areas of health care, and it is a first generation terminology system. The primary aim of KVÅ is that it should consist of procedures done in direct contact with the patient, i.e. not all procedures done in health care [15].

SNOMED CT is an international terminology for the EPR, and was formed in 1999 by the convergence of SNOMED RT and the United Kingdom's Clinical Terms Version 3 (formerly known as the Read Codes), originally developed for primary care [16,17]. SNOMED CT is considered to be a national interdisciplinary terminology by the Swedish National Board of Health and Welfare [18], and it is regarded as evolving towards a third generation terminology system [19]. The Swedish National Board of Health and Welfare has mapped (linked) the non-surgical part of KVÅ to SNOMED CT. Such a mapping may allow organisations to use two or more PCS together; for example, in order to compare statistics with different versions of terminology systems, and to achieve system interoperability and data standardisation [20].

Usage of terminology in information systems requires decisions on how the terminology should fit into the information structure, for example with information standards. This process is often called terminology binding [21].

The use of a PCS involves the structured use of clinical data as the source for determining the appropriate code assignment within a terminology or classification [22]. The reason for introducing procedure coding can be perceived as unclear by health care personnel and can also result in additional work [23,24]. Therefore it is important to gain more knowledge about how procedure coding could be performed and its potential effect on clinical work. How terminology systems such as SNOMED CT can be used by GPs or other health care professionals to code and describe procedures in primary care is still unclear. GPs play a key role in primary care, and we therefore chose to focus on procedures documented by GPs in this initial study of documented procedures. More specifically, we have studied how Swedish GPs document procedures in primary care and to what extent two different terminology systems, KVÅ and SNOMED CT, can be used to structure this documentation.

### Aims

The overarching aim of this study is to explore procedure coding systems in primary care and to describe the implications of their use for health care practice and research.

The detailed objectives of this study are:

• to describe procedures documented by GPs in Swedish EPRs

• to describe and compare how the content in the different terminology systems KVÅ and SNOMED CT covers the procedures' content as they are documented by GPs

• to describe and compare the degree of concordance between the documented procedures and the two terminology systems

• to provide recommendations for future use of standardized terminology systems for research, statistics and quality assurance

## Methods

Two hundred anonymised record entries, documented during 2005, were randomly selected from a research database containing 11 000 000 record entries from EPRs collected from 24 primary care centres in the Skaraborg area in Sweden from 1991 to October 2006. All existing professions and types of contacts were included. Two hundred record entries were considered a representative sample for manual coding and for fulfilling the aims of the study. The year 2005 was chosen for sampling because it was the most recent complete year of collected EPR data in the database. The intention was to retrieve GP notes only. However, the profession title had been removed from all clinicians during the anonymisation step. During the randomized selection, record entries from GPs were identified in a probability based process using identification numbers of the clinicians and contact types for their record entries. No terminology system had been used to code procedures in the record entries.

### KVÅ

KVÅ was chosen for this study because it is used in Swedish health care and has been proposed to be mandatory for reporting procedures in primary care. The version of KVÅ that was used was available in July 2009 and had 9 329 coded categories. Every category belongs to one of eight chapters and also to one of 23 sections. Otherwise the 9 329 categories do not have a hierarchical structure with rubrics as in the International Statistical Classification of Diseases and Related Health Problems (ICD-10) [15].

### SNOMED CT

SNOMED CT was chosen because it is a national interdisciplinary terminology and was recently translated into Swedish for use as a resource together with traditional classifications. The SNOMED CT versions used were the international version from July 2009 with approximately 300 000 concepts and the Swedish versions developed during 2009 and 2010. Every active SNOMED CT concept (except the SNOMED CT "Root concept") has at least one *Is a *relationship to a supertype ("Parent") concept. A concept in SNOMED CT can have more than one *Is a *relationship to "parent" concepts, which creates a poly-hierarchical structure [25].

### Identifying concepts

Content analysis is a research method used to make inferences from textual data by grouping together similar types of utterances and ideas [26]. A framework for using content analysis in identifying meaningful concepts in free text in medical records has been developed for linking qualitative texts to the International Classification of Functioning, Disability and Health-Children and Youth Version (ICF-CY) [27]. For the purpose of this study, we used a method influenced by content analysis to identify meaningful concepts that were explicitly documented as a procedure in the context of each record entry. We chose to analyse only the manifest content, i.e. the written context, not the content that was left out.

Health care activity has been defined as activity performed for a subject of care with the intention of directly or indirectly improving or maintaining the health of that subject of care [28]. Our definition of a procedure was: an intentional procedure done by the responsible GP as a part of directly or indirectly improving or maintaining the health of the patient.

### Coding process

A manual coding process was carried out. The coders were two of the authors (AV and MH), one with experience with SNOMED CT coding and one with no experience with SNOMED CT. None of the coders had previous experience in coding with KVÅ. A panel of experts was consulted for issues that needed clarification both regarding the identified procedures and the coding of procedures. In the panel of experts (SK, GHN and LES), two were GPs (LES, GHN), and two had experience in classification coding (LES, GHN). One (GHN) was an expert reviewer in the project of translating SNOMED CT to Swedish and one (SK) was an expert in medical informatics. Certain questions concerning KVÅ were addressed to an expert at the Swedish National Board of Health and Welfare.

We browsed for concepts in SNOMED CT using the freeware browser CliniClue and the translation platform Health Term used for translating SNOMED CT to Swedish, with the aim of finding health care procedures on the most detailed level possible [29,30]. Two researchers (AV and MH) analysed the record entries in eight sequences, with 20-40 record entries in each sequence. The researchers first examined the record entries of each sequence independently and then together in order to agree on identification and encoding of the procedures. If the researchers disagreed, the panel of experts was consulted to reach a decision on how to interpret and code a procedure. When procedures in all record entries were identified and coded, a quality review was carried out by one of the coders (AV) to find and eliminate inconsistencies in the coding, which were also discussed with the expert panel.

### Coding rules

Coding rules were set up before and during the process. Examples of coding rules were to allow coding to more than one concept for a procedure, and not to code prescribed substances or the exact time when a procedure should be done or was done. Prescriptions that consisted of several substances or drugs in one record entry were only coded once. Procedure concepts in SNOMED CT normally mean that they have actually occurred [25]. Representation of a clinical meaning using a combination of two or more concepts with SNOMED CT (post-coordination) was only used to specify the degree of completion, or states, of a procedure, as well as its various future states prior to its being initiated or completed; for example, if the procedure was "planned" [25]. These states illustrate that procedures have dynamic characteristics that can change in a clinical process.

### Assessment

An assessment of the concordance between each coded procedure-category/concept pair was performed. A pair is either a procedure coded with a category in KVÅ or a procedure coded with a concept in SNOMED CT. The degree of concordance of the procedure with the coded category in KVÅ was assessed, as well as the degree of concordance of the procedure with a concept in SNOMED CT. If a procedure was not coded with a category or a concept, an assessment could not be done. The assessment scale used is seen in Table 1.

**Table 1:** Assessment scale

Code	Text
1	complete match between source and target
2	source is more specific than target
3	source is more general than target
4	source and target are partly overlapping

Assessment scale used for assessing concordance between coded

procedure-category/concept pairs. Source is procedure in record entry; target is category/concept in the Classification of Health Interventions (KVÅ) or SNOMED CT.

### Aggregation and abstraction

In order to describe how the procedures that were coded with SNOMED CT were presented at a detailed and at a more general level, we used the poly-hierarchic structure and the defining *Is a *relationships of SNOMED CT and generated computer-assisted aggregations using the algorithm described and exemplified below. Each of the chosen SNOMED CT concepts was extracted together with its ancestors (all generic concepts) in SNOMED CT including the number of times they had been selected in the coding process. Procedure concepts in the record entries that could not be coded with KVÅ were manually grouped to more general procedure concepts using a method influenced by content analysis to describe what types of procedures were not adequately covered by KVÅ.

The regional ethics review board in Gothenburg approved the study (2005 no.494-05).

## Results

### Procedures documented by GPs

There were 417 procedures found in the 200 EPR record entries. The numbers of procedures identified in each record entry are shown in Table 2. Most commonly, only one procedure was present (43.5%) in each record entry. In 10.0% of the record entries no procedure was found. With a few exceptions, medical history taking and physical investigation procedures to assess health status were usually not documented as procedures in the record entries.

**Table 2:** Procedures in record entries

Procedures (n)	Record entries with none or a certain number (%) of procedures
0	20 (10.0)
1	87 (43.5)
2	31 (15.5)
3	25 (12.5)
4	16 (8.0)
5	10 (5.0)
6	5 (2.5)
7	3 (1.5)
8	1 (0.5)
9	1 (0.5)
10	0 (0.0)
11	1(0.5)

Procedures found in the 200 record entries

### Procedures not coded

Sixteen procedures (3.8%) could not be coded with any terminology system. They were most commonly related to information sharing; for example, procedures that described sending letters to other caregivers, or the patient, with different content, or procedures concerning a service for customized dosage packages of drugs called ApoDos [31]. Eighteen procedures (4.3%) could not be coded with SNOMED CT and 95 procedures (22.8%) could not be coded with KVÅ. The procedures not coded with KVÅ were grouped to more general procedure concepts (Table 3). Planned or booked future treatments or investigations, regardless of type, could not be coded with KVÅ.

**Table 3:** Procedures not coded with the Classification of Health Interventions (KVÅ) grouped to general concepts

Grouped procedures	Number	Example
Prompts the patient or family member to get in touch if necessary	31	Returns if she does not improve
Planned or booked future care	29	Schedules for an appointment for gynecological examination
Order, print or send patient documents to patient or other care-giver	18	A copy to nurse at nursing home
Expectancy	8	Besides that, expectancy
Recommendation to the patient to contact other caregiver	4	I recommend a direct contact with family counseling
Decision about delivery method for medication from Pharmacy	3	ApoDos renewal
Not possible to categorize	2	Decision about a procedure that should not be done

### Description and comparison of content

The content coverage differed extensively between the two terminology systems. There were 399 (95.7%) procedures in the record entries that could be coded with SNOMED CT, compared to 322 (77.2%) that could be coded with KVÅ. The procedures were coded with 148 different SNOMED CT concepts and 36 different KVÅ categories.

**Figure 1:**
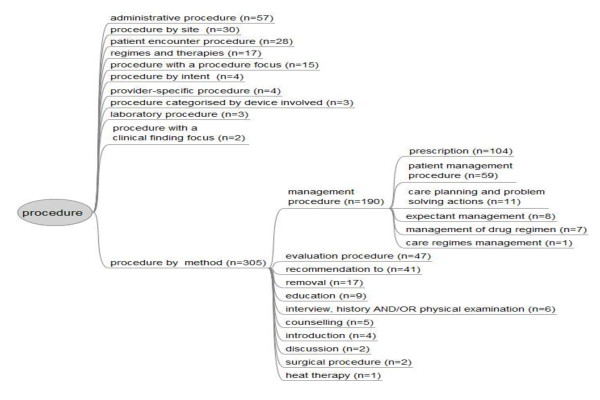
**Procedures in SNOMED CT found in record entries**. This figure shows the distribution of procedures from the record entries from the top node "Procedure" to the nearest subtype or child-concepts, and the distribution in the "Management procedure" concept-tree. "Procedure by site", "Procedure by method" and "Administrative procedure" are all examples of the nearest subtype to the Procedure concept. "Prescription" is a subtype of "Management procedure".

Figure 1 shows the distribution of procedures from the top node concept "procedure" in SNOMED CT, and the most common procedure "Procedure by method" (n = 305). The number of coded procedure-concept/category pairs differed between the terminology systems since some procedures were not possible to code and others were coded with more than one concept/category; SNOMED CT (422 pairs) and KVÅ (323 pairs). The procedures coded with KVÅ-categories at a 1% cut-off level are shown in Table 4. With regard to the above, "Referrals" can serve as an example. "Referrals" in KVÅ were coded with "Referral NOS" (not otherwise specified), i.e. it was not possible to code the type of recipient in KVÅ. Referrals were described with almost the same frequency in KVÅ (n = 55) as in SNOMED CT (n = 58), whereas in SNOMED CT referrals were coded with 19 different types of recipients; for example, the radiology department (n = 15), the surgical service (n = 9), and professionals in the medical service (n = 8). Thirty-two (16.0%) of the encounters had one or more than one referral to another department, excluding radiology.

**Table 4:** Classification of Health Interventions (KVÅ) categories used for coding

Classification of Health Interventions category	Number (%)
Prescription of drug	126 (30.2)
Referral NOS	55 (13.2)
Information/education about examinations and treatments	37 (8.9)
Certificate, simple	12 (2.9)
Information and counseling with the patient by post	13 (3.1)
Information/education about self-care program	14(3.4)
Specimen collection NOS	7 (1.7)
Information and counseling with the patient by phone	8 (1.9)
Other specified specimen collection	7 (1.7)
Certificate, extensive	5 (1.2)
Obtaining advice from other health care personnel NOS	4 (1.0)

KVÅ-categories used for coding at a 1% cut-off level, shown in number and % of procedures in the record entries.

Another example is procedures concerning drugs and medication, which had the following frequencies in the record entries: KVÅ (n = 126) and SNOMED CT (n = 142). Procedures coded with KVÅ were all coded to the category "Prescription of drugs" (n = 126) with the definition "Measures during the initiation, evaluation and release of drugs that include considerations together with the patient regarding prescriptions, written instructions, evaluation of efficacy and side effects". Procedures were coded with 21 different concepts in SNOMED CT; for example, "Prescription of drug" (n = 94), "Recommendation regarding when to take drug" (n = 9) and "Prescription renewal" (n = 6).

### Description and comparison of the degree of concordance

**Figure 2:**
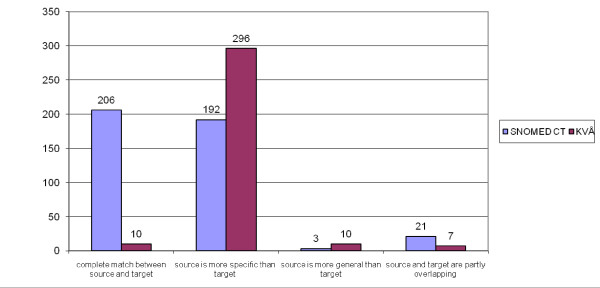
**A comparison of the degree of concordance between procedure- concept/category pairs with SNOMED CT and the Classification of Health Interventions (KVÅ)**. The figure shows the number of procedure- concept/category pairs coded to SNOMED CT and the Classification of Health Interventions (KVÅ) assessed to a value in the assessment scale. "Source" is procedure in record entries and "target" is category/concept in KVÅ or SNOMED CT.

A comparison of the degree of concordance between the procedure- concept/category pairs coded with SNOMED CT and with KVÅ is shown in Figure 2. The procedures coded with KVÅ were assessed as having a much lower concordance than those coded with SNOMED CT. In KVÅ, 10 procedure-concept/category pairs were a complete match compared to 206 to SNOMED CT. Almost all of the procedures identified in the text were regarded as more specific than the categories found in KVÅ; for example, "Specimen collection, NOS". Of the procedures coded with SNOMED CT, 48.1% were considered as more specific than the concept found. Procedures that involved relatives, for example information or instructions to a relative, were difficult to find in SNOMED CT and were assessed at a lower level of concordance in SNOMED CT than in KVÅ. An example of assessment of procedures that differed between the two terminology systems is shown in Table 5.

**Table 5:** Different assessments of coded procedure-category/concept pairs

Procedure	SNOMED CT concept	SNOMED CT assess-ment	KVÅ-category	KVÅ assess-ment
Referral to outpatient ophthalmology clinic	Referral to ophthalmology service	1	Referral NOS	2
Drug treatment stopped	Drug treatment stopped-medical advice	1	Prescription of drug	2
Calls the patient's mother and asks her to collect a new urine sample	Informing next of kin	4	Information and counselling with next-of- kin by telephone	2
Alter drug dosage	Prescription dose change	1	Prescription of drug	2

Examples of different assessments of procedure-category/concept pairs coded with SNOMED CT and Classification of Health Interventions (KVÅ)

When the procedures were explicitly documented with dynamic characteristics or states, they were post-coordinated in SNOMED CT as follows: "carried out" (n = 32), "planned" (n = 31), "requested" (n = 10), "needed" (n = 5), "rescheduled" (n = 1) and "not done" (n = 1). Despite the post-coordinations, reasoning about further contacts or future plans and treatments related to possible changes in the patient's health condition is not sufficiently captured in the coding by either terminology system. An example is "If still not well, or if new recurrence (occurs), referral to xxx department for investigation" (specified department and investigation are left out). This is also the case for planned drug prescriptions for the short or long term that were described in the record entries; for example, "Initially 4 tablets a day, reduce (medication) after the patient notices an improvement".

## Discussion

In summary, the 417 procedures found in the 200 EPR entries were coded with 36 different KVÅ categories and 148 different SNOMED CT concepts. Of the procedures, 22.8% could not be coded with any KVÅ category and 4.3% could not be coded with any SNOMED CT concept. In SNOMED CT, 206 procedure-concept/category pairs were assessed as a complete match compared to 10 in KVÅ.

### Procedures documented by GPs

Referrals to other caregivers were found in 13.3% of the encounters in general practice in Australia during the period 2009-2010, and these included referrals of reproductive health problems and excluded Radiology referrals [7]. In a Nordic study this figure was 8-9% [6]. In our study we found that 16.0% of the encounters had at least one referral to other caregivers, excluding referrals to Radiology departments. We also found that 48 procedures regarding recommendations to the patient, counselling and discussion were found in 200 encounters compared to 29.6 in 100 encounters in 2009-2010 [7] and 25.5 in encounters in 1990 in Australia [32]. Advice and support probably occur as an inherent part of most doctor-patient encounters, as they are an inherent part of general practice [32].

### Description and comparison of content

We had reason to expect that SNOMED CT would cover the GPs' primary care domain well, as one of the terminology systems that was merged into SNOMED CT was originally developed for primary care [16,17]. This expectation was confirmed, as SNOMED CT had much higher content coverage of procedures documented by GPs in primary care than KVÅ.

Low reliability between coders and coding inaccuracy have previously been documented [11,33]. However, medium to high reliability, correctness and completeness have also been reported regarding primary care data [3,34]. Our method was designed to reach a high level of correctness regarding finding all relevant procedures in the record entries, as well as to code the procedures on a high level of detail. As we expected, coding to SNOMED CT with this ambition was both difficult and time-consuming when the optional concepts found were not a complete match. This raised new questions about the procedures identified in the EPR. The coding and assessment tasks meant that analysis of the text had to proceed interactively until a decision was taken. Also, the differences between similar concepts in SNOMED CT could be difficult to understand and needed to be analysed.

We discovered early in the coding process that it was not possible to code the procedures found to KVÅ on a high level of detail, which was not expected. Coding to the general categories in KVÅ did not raise the same questions about the meaning of the procedures in the EPRs and the meaning of categories found. Instead, more time was spent in trying to find possible categories in order to reach a higher level of content coverage.

### Description and comparison of the degree of concordance

The procedures coded with KVÅ were assessed as having much lower concordance than the coding to SNOMED CT, which was expected given the size of the terminology systems. Taking into account that 48.1% of the procedures coded with SNOMED CT were considered more specific than the concept found, combining concepts (post-coordination) could have improved the concordance with SNOMED CT. The reason for not using post-coordination more than we did was that the focus of our study was on what was actually done (the procedures). Post-coordination could possibly have been used in this study to specify the reasons for examinations, the body-parts examined, and the drugs prescribed, if such information was wanted. Post-coordination could also be used to indicate that procedures were not performed on the patient. The structure of a terminology system such as SNOMED CT, increased the possibilities for multipurpose data aggregation. The first generation terminology system structure of KVÅ required manual grouping of categories, which is a method that could lead to arbitrary, non-comparable groups and is prone to error [35]. This is a disadvantage of every first generation terminology system such as, for example, ICD-10. The possibility of aggregating data through the SNOMED CT hierarchy means that concepts on the same general level as in KVÅ can also be found in SNOMED CT and used for statistical purposes. This implies that SNOMED CT can be used in primary care for the same purposes as KVÅ. However, certain areas need to be better covered such as information or instructions to relatives.

### Limitations

The number of record entries (200) is limited, and our figures therefore have to be considered as estimates. The record entries were randomly selected from 11 000 000 record entries in the database and therefore constituted a representative sample. The identification of GPs responsible for the record entries had to be done through types of encounters. However, measures were taken to ensure that the record entries used were in fact documented by GPs. The coding performed was secondary coding, based on the text that was written, and it was done by persons other than the GPs responsible for the documentation in the record entries. This may have resulted in minor differences compared to primary coding by GPs.

### Implications for health care and research

In a review that included earlier versions of SNOMED over a 40-year period, it was reported that studies of SNOMED in clinical practice were scarce [36]. Most uses of SNOMED CT remain basic, and do not capitalize on the rich semantics of the terminology [37]. However, SNOMED CT was approved as a new information standard in the UK and may be used as the clinical terminology within all electronic patient-level communications within the healthcare environment [38], thereby further increasing the importance of studies focusing on the clinical use of SNOMED CT.

Coding of procedures can both clarify and reduce information. If coding is performed by health care professionals when documenting in the patient record, the process used by the documenting GP/health care professional in deciding about a procedure code could possibly lead to more exact expressions about what procedures are done or planned for the patient. Coding can be done as secondary coding by personnel other than the GP or other health care professionals. In our study it was difficult when coding to decide between "possible" procedures and procedures decided upon, and reasoning regarding alternative courses of action depending on a future health condition of the patient. The text sometimes included reasoning about treatment options that was difficult to analyse regarding what decisions were finally made. On the other hand, some information reduction cannot be captured by coding; for example, in the context of what procedures should be done under certain conditions.

Record entries written by GPs in primary care contain a broad range of procedures that are often coded with different terminology systems [6]. What is to be coded and what constitutes a meaningful level for recording procedures depends on the purpose of the coding. Primary care in Sweden accounts for 17% of the net costs of health care, and yet there is little knowledge about the procedures performed by different professions. However, coding of procedures is time-consuming, and is an additional administrative task over and above practical care [23,24]. Collection and secondary use of procedures as coded data should have clear clinical or administrative purposes such as information for other caregivers or professions about planned procedures, as a basis for reimbursement, or for studying certain areas of interest to evaluate procedures related to health problems.

Coding must be supported with suitable tools and integrated in the EPR design with appropriate terminology integration or binding to the information model, which includes support for documenting the dynamic states of procedures in the clinical process. If SNOMED CT is to be used in procedure coding, it is necessary for the end user to have support with the coding. This would include a subset of procedure concepts available for the domain, and/or tools for text reading with semiautomatic primary or secondary coding suggestions. There is also a lack of tools for aggregation and presentation of data based on SNOMED CT coding in order to take advantage of the SNOMED CT concept model. Our experience with the process of coding with post-coordination and reuse of post-coordinated concepts in SNOMED CT is that the procedure is complicated and requires supportive tools if done by end users.

In choosing a PCS, the degree of content coverage is an important factor [13]. Previous attempts to introduce procedure coding in primary care in Sweden have not been successful. The reasons for this remain unknown, but the poor content coverage described in this study needs to be addressed in the future to potentially increase acceptance and usability of PCS. The reasons for using a PCS can vary and can include clinical, statistical or reimbursement purposes. This means that a PCS should have a flexible design and be functional in different settings. It is important for terminology systems to be able to be used in everyday multi-professional environments. Thus procedures documented by different health care professionals in primary care, such as nurses and physiotherapists, need to be explored in relation to KVÅ and SNOMED CT. SNOMED CT could be of benefit in the aggregation and interpretation of epidemiological statistics when analysing data from primary care, as well as in following up clinical data and in quality assurance.

## Conclusions

Procedures documented by GPs are present in nearly all EPR entries, and the spectrum of procedures documented by GPs in primary care is wide. SNOMED CT covered almost all of the procedures' content. KVÅ covered the procedures to a lesser extent and with a much lower degree of concordance. SNOMED CT is a more flexible terminology system that can be used for different purposes for procedure coding in primary care. Our findings imply that to support clinical procedure coding, a number of prerequisites such as terminology binding, shortlists of procedures and coding support in the EPR need to be fulfilled.

## Competing interests

MN is a member of IHTSDO's Technical Committee and is Sweden's Leading Technical Contact with IHTSDO. GHN has participated in the Swedish translation of SNOMED CT.

## Authors' contributions

AV: participated in the design of the study, coded the data, participated in the analysis and drafted the manuscript. MH: participated in the design of the study, coded the data, participated in the analysis and in writing the manuscript. MN: participated in creating the EPR database, designed, implemented and ran the algorithms for the analysis, and wrote about those parts in the article. LES and SK: participated in the design of the study, helped with the analysis, and participated in writing the manuscript. PH and UL: were responsible for creating the database with EPR-data, and participated in writing the manuscript. GHN: participated in the design of the study, participated in the analysis and wrote parts of the manuscript. All authors read and approved the final manuscript.

## Pre-publication history

The pre-publication history for this paper can be accessed here:

http://www.biomedcentral.com/1471-2296/13/2/prepub
